# Long‐Term Effects of Sglt2 Deletion on Bone and Mineral Metabolism in Mice

**DOI:** 10.1002/jbm4.10526

**Published:** 2021-07-06

**Authors:** Claire Gerber, Xueyan Wang, Valentin David, Susan E. Quaggin, Tamara Isakova, Aline Martin

**Affiliations:** ^1^ Division of Nephrology and Hypertension, Feinberg School of Medicine Northwestern University Chicago IL USA; ^2^ Center for Translational Metabolism and Health, Institute for Public Health and Medicine Northwestern University Chicago IL USA; ^3^ Feinberg Cardiovascular and Renal Research Institute Northwestern University Chicago IL USA

**Keywords:** BONE, CALCIUM, GLYCOSURIA, PHOSPHATE, SGLT2

## Abstract

Sodium‐glucose cotransporter 2 (SGLT2) inhibitors improve kidney and cardiovascular outcomes in patients with type 2 diabetes mellitus (T2DM). However, bone fragility has emerged as a side effect in some but not in all human studies. Because use of SGLT2 inhibitors in humans affects mineral metabolism, we investigated the long‐term effects of genetic loss of Sglt2 function on bone and mineral metabolism in mice. *Slc5a2* nonsense mutation in Sweet Pee (SP) mice results in total loss of Sglt2 function. We collected urine, serum, and bone samples from 15‐week‐old and 25‐week‐old wild‐type (WT) and SP mice fasted from food overnight. We measured parameters of renal function and mineral metabolism and we assessed bone growth, microarchitecture, and mineralization. As expected, 15‐week‐old and 25‐week‐old SP mice showed increased glucosuria, and normal kidney function compared to age‐matched WT mice. At 15 weeks, SP mice did not show alterations in mineral metabolism parameters. At 25 weeks, SP mice showed reduced fasting 24‐hour urinary calcium excretion and increased fractional excretion of phosphate, but normal serum calcium and phosphate, parathyroid hormone (PTH), vitamin D (1,25(OH)_2_D), and fibroblast growth factor (FGF23) levels. At 25 weeks, but not at 15 weeks, SP mice showed reduced body weight compared to WT. This was associated with reduced femur length at 25 weeks, suggesting impaired skeletal growth. SP mice did not show trabecular or cortical bone microarchitectural modifications but showed reduced cortical bone mineral density compared to WT mice at 25 weeks. These results suggest that loss of Sglt2 function in mice in the absence of T2DM does not alter regulatory hormones FGF23, PTH, and 1,25(OH)_2_D, but may contribute to bone fragility over the long term. Future studies are required to determine how loss of Sglt2 function impacts bone fragility in T2DM. © 2021 The Authors. *JBMR Plus* published by Wiley Periodicals LLC on behalf of American Society for Bone and Mineral Research.

## 1 Introduction

Type 2 diabetes mellitus (T2DM) affects 29 million Americans and is a leading cause of blindness, kidney failure, amputations, and cardiovascular disease.^(^ ^1^, ^2^ ^)^ T2DM increases fracture risk,^(^ ^3^, ^4^, ^5^, ^6^, ^7^, ^8^, ^9^, ^10^ ^)^ which further contributes to morbidity, mortality, and costs.^(^ ^11^, ^12^, ^13^, ^14^ ^)^ Multiple factors contribute to the increased susceptibility to bone fractures in patients with T2DM, including abnormal bone mineral metabolism, poor bone quality due to poorly managed glucose and insulin levels, increased risk of falls, and adverse side effects of various drugs.^(^ ^15^ ^)^

Sodium–glucose cotransporter 2 (SGLT2) inhibitors bind to the SGLT2 transporter and prevent the reabsorption of glucose and sodium in the renal proximal tubule, thereby promoting urinary glucose and sodium excretion.^(^ ^16^, ^17^, ^18^ ^)^ This new class of glucose‐lowering agents improves cardiovascular outcomes, while also lowering systolic blood pressure and body weight and having beneficial effects on kidney function in patients with T2DM.^(^ ^19^, ^20^ ^)^ Despite all the promising clinical outcomes, use of certain SGLT2 inhibitors, including canagliflozin and dapagliflozin, showed an association with increased risk of bone fractures in some but not in all studies.^(^ ^21^, ^22^, ^23^, ^24^ ^)^ Although the exact reasons for the differing observations remain uncertain, detailed physiologic human studies have linked SGLT2 inhibitors with altered mineral metabolism.^(^ ^21^ ^)^ A study of healthy volunteers treated for 5 days with 300 mg/d of canagliflozin showed that SGLT2 inhibition resulted in increased calciuria, reduced urinary phosphate loss, and increased serum phosphate.^(^ ^25^ ^)^ In a T2DM population, 10 mg/d dapagliflozin induced a significant increase in serum phosphate, parathyroid hormone (PTH), and fibroblast growth factor 23 (FGF23) over a 6‐week treatment period.^(^ ^26^ ^)^ To date, the effects of long‐term inhibitor treatment are still unclear. In a study performed on 50‐week‐old “Jimbee” mice that displayed Sglt2 loss‐of‐function, no significant change in FGF23 were observed, but the mutation did negatively impact growth of the femur and resulted in lower tissue mineral density in cortical and trabecular bone.^(^ ^27^ ^)^ Overall results from previous studies in humans and animals provide inconsistent findings on how loss of SGLT2 function or inhibition of SGLT2 action affects mineral metabolism and bone health.

To understand the impact of Sglt2 inhibition on mineral and bone metabolism in settings of normal kidney function, we utilized the previously characterized Sweet Pee (SP) mouse model containing a nonsense mutation in the *Slc5a2* gene that results in loss of Sglt2 function.^(^ ^28^ ^)^ We anticipated that loss of Sglt2 function in the renal proximal tubule would lead to early alterations in urinary calcium and phosphate excretion that could elicit detectable and sustained changes in regulatory hormones and possible alterations in bone phenotype at a later time point. Therefore, we performed a longitudinal study to characterize how sustained glucosuria impacted mineral metabolism parameters, including serum and urinary calcium and phosphate, and serum FGF23, PTH, 1,25 dihydroxyvitamin D (1,25(OH)_2_D), at 15 and 25 weeks of age, and skeletal phenotype in mature 25‐week‐old mice. We hypothesized that loss of Sglt2 function in a genetic mouse model would lead to (i) early alterations in mineral metabolism including increased urinary calcium excretion, decreased urinary phosphate excretion, decreased serum calcium, and increased serum phosphate; (ii) sustained elevation in PTH and FGF23; and (iii) reduced cortical and trabecular bone mass in SP mice compared to wild‐type (WT) mice.

## 2 Materials and Methods

### 2.1 Animal studies

SP mice were previously generated by N‐ethyl‐N‐nitrosourea (ENU) mutagenesis and identified to contain a point mutation in the *Scl5a2* gene leading to mice that do not express Sglt2, as described.^(^ ^28^ ^)^ All WT and SP mice were maintained on a C3H genetic background. WT and SP male littermate mice were fed a standard rodent chow ad libitum and were harvested at 15 and 25 weeks (*n* = 10/group). Animals were housed in the Northwestern Chicago Animal Facility on a 12‐hour light–dark cycle. All animal studies were conducted in accordance with the Northwestern University Institutional Animal Care and Use Committee. Prior to harvest, mice were housed in individual metabolic cages, in which they were fasted from food, but not water, for 20 to 24 hours.^(^ ^29^, ^30^ ^)^ All urine produced during this period was collected and weighed. Total urine volume was standardized to 24 hours. Serum samples were collected by intracardiac exsanguination. Serum and urine samples were stored at −80°C until analyzed. Bone samples were also harvested at euthanasia.

### 2.2 Serum and urine biochemistry

Serum intact and total FGF23 levels were measured using commercially available mouse iFGF23 (recognizes intact FGF23 only) and cFGF23 (recognizes both intact and C‐terminal FGF23 peptides) ELISA assays (Immutopics, Carlsbad, CA, USA). Serum intact PTH was measured using the mouse PTH 1‐84 ELISA assay (Immutopics) and serum 1,25(OH)_2_D was measured by immunoassay (Immunodiagnostic Systems, Gaithersburg, MD, USA). Blood urea nitrogen (BUN), serum and urine calcium, phosphate, creatinine, and urinary albumin were measured using colorimetric assays (Pointe Scientific, Canton, MI, USA). Serum potassium was measured using turbidimetric assay (Stanbio, Boerne, TX, USA). Serum ketones were measured using the Precision Xtra ketone monitoring system (Abbott, Alameda, CA, USA). Urinary glucose was measured using Accu‐Chek Aviva glucometer (Roche, Indianapolis, IN, USA).

### 2.3 Cell cultures

We prepared bone marrow stromal cells (BMSCs), cortical and calvaria bone osteoblasts from 30‐week‐old WT and SP mice according to a previously described protocol.^(^ ^31^ ^)^ We plated 10 × 10^4^ cells per well and cultured for 3 weeks in osteoblast‐differentiating medium (α‐minimal essential medium, 10% fetal bovine serum, 10 U/mL penicillin, 100 μg/mL streptomycin, 10 mmol/L β‐glycerophosphate, and 50 μg/mL ascorbic acid; Sigma‐Aldrich, St Louis, MO, USA) prior to collection.

### 2.4 RT‐PCR

We isolated total RNA from kidney and tibia samples harvested at euthanasia and from primary osteoblasts cultures using TRI reagent and synthesized first‐strand cDNA (iScript cDNA Synthesis Kit; Bio‐Rad Laboratories, Hercules, CA, USA). We used the iCycler iQ real‐time PCR detection system, iQ SYBR Green supermix (Bio‐Rad Laboratories) for real‐time quantitative PCR analysis. The threshold of detection of each gene expression was set at optimal reaction efficiency. The expression was plotted against a standard dilution curve of relative concentration, normalized to ribosomal protein L19 (Rpl19) expression in the same sample and expressed as fold change versus WT.

### 2.5 Three‐dimensional micro–computed tomography

Whole femurs from 25‐week‐old mice were scanned using micro–computed tomography (μCT) with μCT40 (Scanco Medical, Brüttisellen, Switzerland) at 10 μm isotropic voxel size, energy level of 55 keV, and intensity of 145 μA to determine bone mineral density.^(^ ^29^ ^)^ Trabecular bone was analyzed within 1 mm of secondary spongiosa of the distal femur underneath the growth plate. The cortical bone was analyzed within 1 mm at the midshaft of each femur. The Gaussian filter was used for all segmented grayscale images.

### 2.6 Bone histology

Seven and 2 days prior to harvest, mice were injected with Alizarin red for intravital staining of the mineralization fronts. We measured femur lengths using a slide caliper. Femurs and tibias were fixed and dehydrated and embedded in methylmethacrylate (MMA). We cut non‐serial 5‐μm longitudinal and cross‐sectional sections (Leica Microsystems Inc., Buffalo Grove, IL, USA) for histological analyses. Sections were either unstained to evaluate double‐labeled mineralizing surfaces, or stained with modified trichrome Goldner to evaluate bone mineralization or with tartrate‐resistant acidic phosphatase (TRAcP) activity staining to evaluate bone resorption according to a previously described method.^(^ ^32^ ^)^

### 2.7 Statistical analyses

We applied a Spearman rank order correlation to test the relationship of 24‐hour urinary glucose with 24‐hour urinary calcium and 24‐hour urinary phosphate. Data are presented as mean ± SEM. We used *t* tests to test statistical differences between groups (Statistica software; Statsoft, Tulsa, OK, USA). All statistical tests were two‐sided, and differences were considered statistically significant at *p* values <0.05.

## 3 Results

### 3.1 Fasted SP mice display high levels of glucosuria compared to WT mice

**Fig 1:**
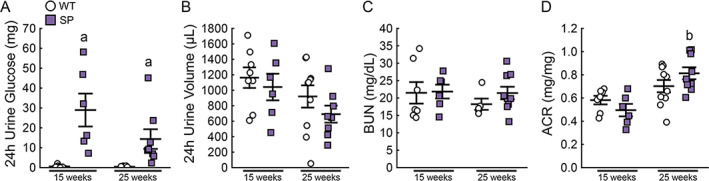
Sglt2 deletion increases urinary glucose excretion. Levels of (*A*) 24‐hour urinary glucose excretion, (*B*) 24‐hour urine volume, (*C*) BUN levels, and (*D*) urine ACR in fasted WT and SP mice at 15 and 25 weeks of age. Values are expressed as mean ± SEM; *n* > 8 male mice/group. *p* < 0.05 versus: ^a^age‐matched WT, ^b^15‐week‐old SP. ACR = albumin to creatinine ratio; BUN = blood urea nitrogen.

It has been well established that SGLT2 inhibition prevents the reabsorption of glucose. Indeed, at 15 and 25 weeks of age, fasted SP mice had markedly higher levels of 24‐hour urinary glucose excretion compared to age‐matched WT mice (Fig. 1*A*, 15‐week 2.9 versus 0.06 g, *p* < 0.05; 25‐week 1.4 g versus 0.04, *p* < 0.05). Even though there was greater magnitude of glucosuria in SP mice compared to WT mice, we did not observe a difference in 24‐hour urine volume between the two groups at either 15 or 25 weeks of age (Fig. 1*B*). We assessed possible signs of dehydration and ketosis and did not detect noticeable changes in water intake (not shown), serum potassium, or serum ketone levels which were similar between WT and SP mice (Figs. S1 and S2). We evaluated the impact of loss of Sglt2 function on kidney function by evaluating serum BUN and albumin creatinine ratio (ACR) in WT and SP mice. SP mice did not display any overt signs of renal injury compared to WT mice at either 15 or 25 weeks (Fig. 1*C*,*D*).

### 3.2 Fasted SP mice do not show increased calciuria or changes in calciotropic hormones compared to WT mice

**Fig 2:**
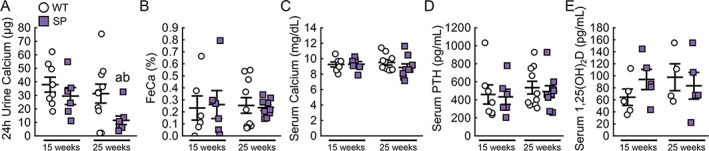
Deletion of Sglt2 does not impact calcium homeostasis. Levels of (*A*,*B*) 24‐hour urine calcium and FeCa, and (*C*–*E*) serum calcium, PTH, and 1,25(OH)_2_D in WT and SP mice at 15 and 25 weeks of age. Values are expressed as mean ± SEM; *n* > 8 male mice/group. *p* < 0.05 versus: ^a^age‐matched WT, ^b^15‐week‐old SP. FeCa = fractional excretion of calcium; PTH, parathyroid hormone.

To test our hypothesis that loss of Sglt2 function has effects on mineral metabolism, mice were further profiled for urinary excretion of calcium and phosphate in addition to hormones that regulate calcium and phosphate levels, including PTH, 1,25(OH)_2_D, and FGF23. Fasted SP mice did not excrete more calcium in 24 hours at 15 weeks of age and excreted significantly less urinary calcium in 24 hours at 25 weeks compared to age‐matched WT mice (Fig. 2*A*, 11.5 versus 31.3 mg, respectively, *p* < 0.05). Evaluation of fractional excretion of calcium determined there was no difference between WT and SP mice at 15 or 25 weeks (Fig. 2*B*). There was no difference in serum calcium between SP mice and WT mice at 15 or 25 weeks of age (Fig. 2*C*). Levels of PTH and 1,25(OH)_2_D were similar between WT and SP mice at 15 and 25 weeks of age (Fig. 2*D*,*E*). mRNA expression levels of 1,25(OH)2D metabolizing enzymes Cyp27b1 and Cyp24a1 in the kidney were also similar between WT and SP mice at 25 weeks of age (Fig. S3).

### 3.3 SP mice show normal phosphate homeostasis compared to WT mice

**Fig 3:**
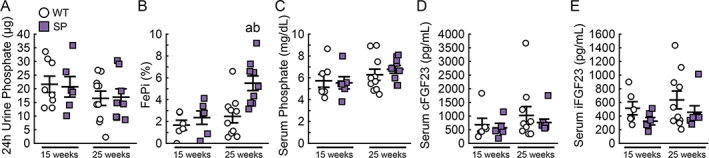
Deletion of Sglt2 does not impact phosphate homeostasis. Levels of (*A*,*B*) 24‐hour urine phosphate and FePi, and (*C*–*E*) serum phosphate, cFGF23, and iFGF23 in WT and SP mice at 15 and 25 weeks of age. Values are expressed as mean ± SEM; *n* > 8 male mice/group. *p* < 0.05 versus: ^a^age‐matched WT, ^b^15‐week‐old SP. cFGF23, total FGF23; FePi, fractional excretion of phosphate; iFGF23, intact FGF23.

Twenty‐four‐hour urinary phosphate excretion was similar between WT and SP mice at 15 or 25 weeks (Fig. 3*A*). Interestingly, SP mice had elevated fractional excretion of phosphate at 25 weeks compared to WT mice (Fig. 3*B*, 5.5 ± 2.0 versus 2.5 ± 1.9%, *p* < 0.05), but had no significant change in serum phosphate levels (Fig. 3*C*). Levels of cFGF23 and iFGF23 were also not different between WT and SP mice at 15 or 25 weeks (Fig. 3*D*,*E*).

### 3.4 Urinary phosphate excretion correlates with urinary glucose excretion

**Fig 4:**
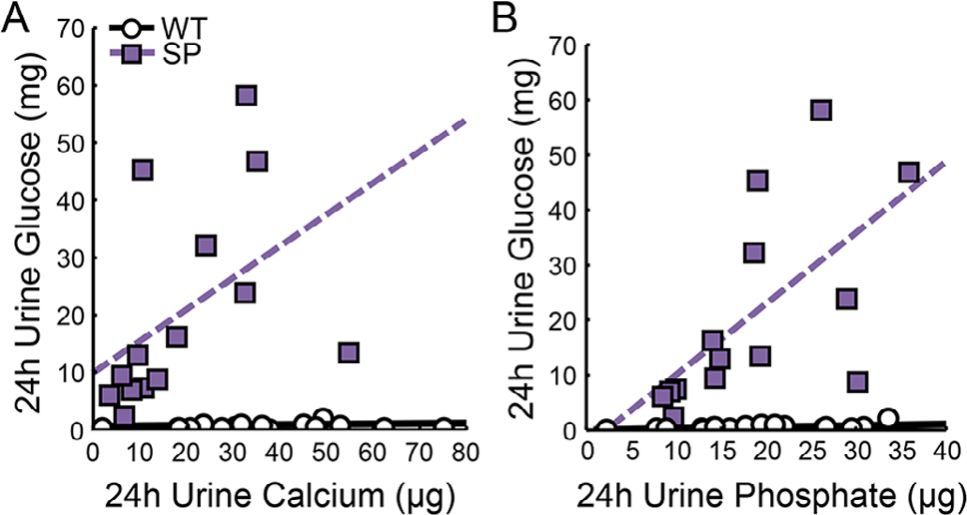
Increased 24‐hour glucose excretion leads to increased 24‐hour phosphate excretion. (*A*,*B*) Spearman correlation between 24‐hour glucose excretion and 24‐hour calcium excretion or 24‐hour phosphate excretion. (*A*) WT *r* = 0.3578 (*p* = 0.159) and SP *r* = 0.5989 (*p* = 0.031) and (*B*) WT *r* = 0.5624 (*p* = 0.015) and SP *r* = 0.7319 (*p* = 0.003).

We combined data from 15‐week‐old and 25‐week‐old fasted mice to evaluate relationships of 24‐hour glucose excretion with 24‐hour calcium and phosphate excretion by Spearman correlation. Twenty‐four‐hour urinary calcium correlated positively with 24‐hour urinary glucose in SP but not in WT mice (Fig. 4*A*, WT *r* = 0.36, *p* = 0.15; SP *r* = 0.59, *p* = 0.03). However, 24‐hour urinary phosphate excretion positively correlated with 24‐hour urinary glucose excretion in both WT and SP mice (Fig. 4*B*, WT *r* = 0.56, *p* = 0.02; SP *r* = 0.73 *p* = 0.003).

### 3.5 Cortical bone mineral density is reduced in SP mice compared to WT mice

**Fig 5:**
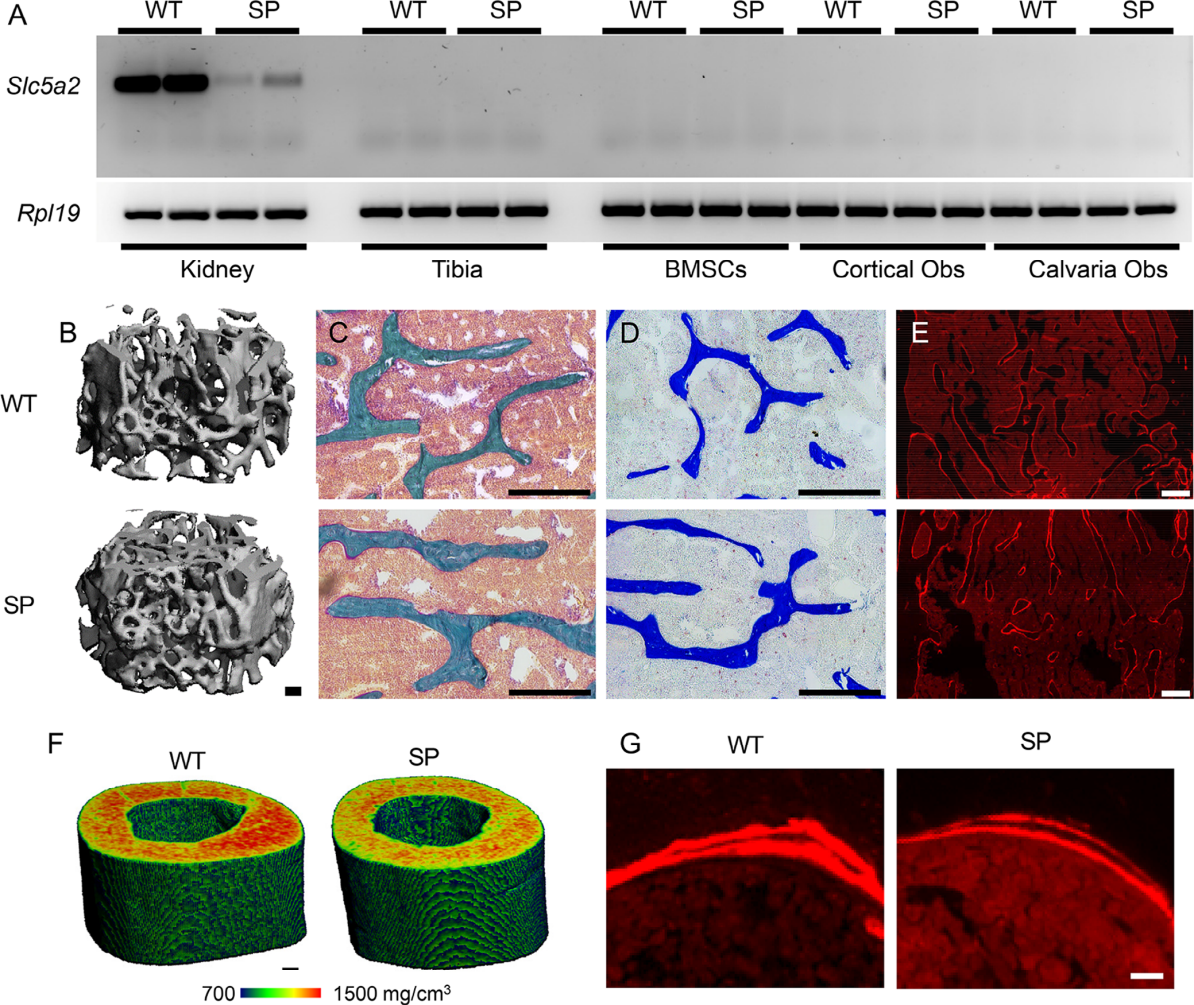
Sglt2 deletion indirectly leads to reduced cortical bone mineral density. (*A*) mRNA expression of Slc5a2 measured by RT‐PCR in whole kidney, whole tibia, and primary cultures of BMSCs, cortical and calvaria bone osteoblasts, isolated from 30‐week‐old WT and SP mice. Expression is normalized by Rpl19 mRNA expression in each sample. (*B*–*G*) Trabecular and cortical bone imaging of femurs from 25‐week‐old WT and SP mice. 3D‐μCT (scale bar = 100 μm) (*B*) and 2D histology analyses by bright‐field microscopy (scale bar = 250 μm) of modified trichrome Goldner (*C*) and TRAcP staining (*D*), and by fluorescent microscopy (scale bar = 200 μm) of (*E*) Alizarin red S double‐labeling of the femur distal metaphysis secondary spongiosa. (*F*) 3D‐μCT analysis of the femur midshaft cortical bone (scale bar = 100 μm). (*G*) The degree of mineralization is represented by the heat map. 2D histology analysis of the tibia midshaft cortical bone by fluorescent microscopy (scale bar = 12.6 μm) of Alizarin red S double‐labeling. μCT = micro–computed tomography; BMSC = bone marrow stromal cell.

SP mice had a similar gross appearance to WT mice. At 15 weeks of age, WT and SP mice showed similar body weight and femur length (Table 1). Consistent with previous report,^(^ ^28^ ^)^ SP mice weighed significantly less at 25 weeks of age and had significantly shorter femurs compared to age‐matched WT control mice (Table 1). To test our hypothesis that Sglt2 deletion leads to reduced bone mass, we measured Slc5a2 expression in kidney and bone, and analyzed the bone phenotype by three‐dimensional (3D)‐μCT and 2D histology in both WT and SP mice at 25 weeks. We confirmed expression of Slc5a2 mRNA in kidney from WT mice. As expected, Slc5a2 mRNA was barely detectable in SP kidneys. Importantly, we did not detect any Slc5a2 mRNA in bone or primary osteoblasts cultures (Fig. 5*A*). There were no significant changes in trabecular bone microarchitecture or bone dynamics between WT and SP mice (Table 2, Fig. 5*B*–*E*) measured by 3D‐μCT and 2D histology. However, we found a significant reduction in 3D cortical bone mineral density (BMD) in SP compared to WT mice (Table 2, Fig. 5*F*), which was not associated with additional changes in cortical bone morphology (Table 2) or cortical bone formation (Table 3, Fig. 5*G*). In aggregate, these data indicate mild defects in cortical bone mineralization, and normal bone remodeling in SP mice. Finally, mRNA expression of markers of growth hormone (GH)/insulin‐like growth factor 1 (IGF1) and Wnt signaling, including Igf1, Sost, Dkk1, and Axin2 mRNA in tibia, was not modified between WT and SP mice at 25 weeks (Fig. S3).

**Table 1:** Growth Parameters in 15‐Week‐Old and 25‐Week‐Old WT and SP Mice

	15 weeks		25 weeks	
Parameter	WT	SP	WT	SP
Body weight (g)	22.7 ± 0.6	21.8 ± 0.5	25.1 ± 0.9 ^*^	21.6 ± 1.3 ^**^
Femur length (mm)	15.0 ± 0.1	15.1 ± 0.1	15.5 ± 0.1 ^*^	15.1 ± 0.1 ^**^

Values are expressed as mean ± SEM; *n* > 5 male mice/group.

* *p* < 0.05 versus 15‐week‐old WT.

** *p* < 0.05 versus 25‐week‐old WT.

**Table 2:** 3D Microtomography Analysis of Femurs from 25‐Week‐Old WT and SP Mice

	WT	SP
Trabecular bone		
3D BMD (mg/cm^3^)	915 ± 24	900 ± 16
BV/TV (%)	24 ± 4	27 ± 5
Tb.N (mm^−1^)	4.5 ± 0.3	4.7 ± 0.5
Conn.Dens. (mm^−3^)	128 ± 19	154 ± 39
Tb.Th (μm)	60 ± 5	60 ± 5
SMI	1.1 ± 0.3	0.8 ± 0.4
Cortical bone		
3D BMD (mg/cm^3^)	1264 ± 14	1240 ± 16 ^*^
Ct.Th (μm)	0.37 ± 0.04	0.36 ± 0.03
CSA (mm^2^)	3.1 ± 0.4	3.2 ± 0.2
Ma.Ar (mm^2^)	1.8 ± 0.4	1.9 ± 0.2
Ct.Ar (mm^2^)	1.4 ± 0.1	1.3 ± 0.1

Values are expressed as mean ± SEM; *n* > 8 male mice/group.

3D = three‐dimensional; BMD = bone mineral density; BV/TV = bone volume fraction; CSA = cross‐sectional area; Ct.Ar = cortical area; Ct.Th = cortical thickness; Conn.Dens = connectivity density; Ma.Ar = marrow area; SMI = structure model index; Tb.N = trabecular number; Tb.Th = trabecular thickness.

* *p* < 0.05 versus age‐matched WT.

**Table 3:** Cortical Bone Formation Parameters From 25‐Week‐Old WT and SP Mice

	WT	SP
MAR (μm/d)	1.17 ± 0.10	1.03 ± 0.09
MS/BS (%)	28.17 ± 4.27	29.20 ± 5.16
BFR/BS (μm^3^/μm^2^/d)	0.34 ± 0.07	0.33 ± 0.07

Quantification was performed by 2D histomorphometry in femur diaphysis. Values are expressed as mean ± SEM; *n* = 5 male mice/group. *p* > 0.05, NS versus WT.

2D = two‐dimensional; BFR/BS = bone formation rate per unit of bone surface; MAR = mineral apposition rate; MS/BS = bone mineralizing surface; NS = nonsignificant.

## 4 Discussion

SGLT2 inhibitors show promise in clinical trials, with recent results demonstrating that SGLT2 inhibitors reduce the risk of cardiovascular events, decrease systolic blood pressure, decrease body weight, and preserve renal function in patients with T2DM with and without chronic kidney disease (CKD).^(^ ^20^, ^23^, ^33^, ^34^ ^)^ Despite the positive results, there is still some inconsistency in terms of possible adverse effects, including the possible increased risk of fracture seen in some of the earlier clinical trials.^(^ ^21^, ^22^, ^23^, ^24^ ^)^ Our aim with this study was to investigate the longitudinal and long‐term impact of loss of Sglt2 function in renal proximal tubules on mineral and bone metabolism in a genetic mouse model in which a frameshift mutation leads to a premature stop signal silencing Sglt2 expression.

Previously, it was shown that unfasted SP mice exhibited high levels of glucosuria, despite unaltered levels of hemoglobin A1c as compared to their WT littermates.^(^ ^28^ ^)^ Dramatic changes in glucose excretion were associated with increased urinary excretion of calcium, magnesium, potassium, creatinine, and urea.^(^ ^28^ ^)^ However, detailed assessments of mineral and bone metabolism in this model have not been performed. We performed a longitudinal analysis of serum phosphate and urinary phosphate excretion, FGF23, PTH, 1,25(OH)_2_D, and bone turnover and mineralization.

For our assessments of mineral metabolism, we housed mice in metabolic cages without access to food for 24 hours, which is the standard method for measuring mineral metabolism parameters.^(^ ^29^, ^30^, ^35^, ^36^ ^)^ Consistent with prior reports, fasted SP mice had increased urinary glucosuria compared to WT mice at 15 and 25 weeks. However, in contrast to a previous study performed in non‐fasted mice,^(^ ^28^ ^)^ we did not detect effects of Sglt2 deletion on fasting urinary calcium excretion at either 15 or 25 weeks. Because fasting decreases the filtered load of glucose in the kidney, our experimental approach has reduced the magnitude of glucosuria (approximately by 25‐fold compared to previous study performed in unfasted mice^(^ ^28^ ^)^) and may have prevented us from detecting changes in urinary calcium excretion. In fact, the significant correlation coefficient of 0.60 between 24‐hour urinary glucose and 24‐hour urinary calcium in SP mice suggests that SP mice likely need to show extremely elevated glucosuria to affect the levels of calciuria. Therefore, in unchallenged SP mice, it is not surprising that in addition to a lack of an effect on urinary calcium excretion at 15 weeks we found normal levels of serum calcium, PTH, and 1,25(OH)_2_D at 15 and 25 weeks. However, case reports describing the mineral metabolism profile of patients with familial renal glucosuria, a hereditary condition characterized by inactivating mutation of SGLT2, have reported hypercalciuria, but PTH levels were also found to be in the normal range.^(^ ^37^, ^38^ ^)^ Thus, even in the context of glucosuria‐induced hypercalciuria in the setting of SGLT2 inhibition, hypercalciuria may not be marked enough to cause detectable alterations in calciotropic hormones.

Contrary to our initial hypothesis that SP mice would exhibit a subtle rise in serum phosphate and excrete less phosphate, we found that there were no differences in 24‐hour urinary phosphate excretion between the two groups. Although fractional excretion of phosphate was significantly elevated in SP mice compared to WT mice at 25 weeks, we did not detect any alterations in serum phosphate nor FGF23. Additionally, we found a positive correlation between 24‐hour urinary glucose and 24‐hour urinary phosphate. Our findings differ from previous human studies that demonstrated decreased urinary phosphate excretion due to increased tubular reabsorption of phosphate in healthy participants and patients with T2DM who were treated with SGLT2 inhibitors.^(^ ^21^, ^25^, ^26^ ^)^ For example, in a single‐blind randomized crossover study, treatment with canagliflozin increased serum phosphate levels by 16% in healthy participants.^(^ ^25^ ^)^ Similarly, in a secondary post hoc analysis of patients with T2DM, dapagliflozin increased serum phosphate by 9%.^(^ ^26^ ^)^ In both healthy and T2DM participants, the rise in serum phosphate contributed to increased FGF23 and PTH and decreased 1,25(OH)_2_D. The increased serum phosphate coincided with a reduced urinary phosphate excretion in both healthy and T2DM participants (4.4% and 12%, respectively).^(^ ^26^ ^)^ Additional studies are needed in SP mice to further clarify effects on phosphate and calcium handling in non‐fasting conditions.

We also hypothesized that changes in mineral metabolism would have adverse effects on bone mass. Compared to age‐matched WT controls, SP mice had significantly shorter femurs and lower body weight than WT mice at 25 weeks of age. Cortical BMD was significantly reduced in SP mice at 25 weeks compared to WT littermates, whereas trabecular microarchitecture and remodeling were similar in SP and WT mice at 25 weeks. Slc5a2 is not expressed in the bone, and kidney function and serum 1,25(OH)_2_D levels were normal in 25 week SP mice, suggesting that other indirect mechanisms are responsible for the reduction in cortical BMD. Although the specific reason remains unclear, we speculate that either mild urinary phosphate loss, or reduced body weight, may have contributed to impaired bone growth and cortical bone mineralization in our model. However, we did not detect any differences in the mRNA expression of markers of GH/IGF1 signaling or Wnt signaling in bone, suggesting that altered growth hormone signaling and mechanical loading contribution to the cortical bone mineralization defect observed in SP mice is minimal. Consistent with our findings, patients with renal glucosuria also display delayed growth and shortened stature.^(^ ^16^ ^)^ In a different model of Sglt2 deletion, 50‐week‐old “Jimbee” male mice also exhibited reductions in body weight and lower tissue mineral density of both cortical and trabecular bone sites.^(^ ^27^ ^)^ In contrast to our study and the “Jimbee” mice, a prior study in healthy mice treated with canagliflozin did not show reductions in cortical or trabecular BMD.^(^ ^39^ ^)^ A bone phenotype was observed in a type 1 diabetes mellitus (T1DM) mouse model treated with canagliflozin. Compared to vehicle treated T1DM mice, T1DM mice treated with canagliflozin had reduced bone formation, increased bone resorption, exaggerated urinary mineral loss, and a secondary increase in FGF23.^(^ ^39^ ^)^ Additional experimental studies in models of T2DM and T2DM with CKD are needed to further define the effect of Sglt2 inhibition on bone health and to assess differences between genetic loss of function and drug‐induced inhibition.

Strengths of our analysis include the evaluation of SP mice at two time points, which helped test the impact of loss of Sglt2 function over an extended period of time on parameters of mineral and bone metabolism. SP mice exhibit high glucose excretion in comparison to WT mice at both 15 and 25 weeks of age. However, upon fasting these animals overnight we did not observe changes in urinary calcium excretion, likely due to limited glucosuria compared to non‐fasted conditions. Additional models in which the filtered load of glucose is a controlled variable are needed. For example, SP mice challenged with varying concentrations of glucose boluses and SP mice challenged with T2DM would perhaps show accentuated glucosuria and increased calcium excretion observed in other studies. Our study is limited to understanding the impact of deletion of Sglt2 in healthy mice. Many T2DM patients develop CKD, which is associated with increased FGF23. The mineral metabolism abnormalities induced by Sglt2 inhibition may exacerbate mineral and bone disorders related to CKD even more, further increasing bone fragility in this already high‐risk population. To address Sglt2 safety profile in CKD, further studies are required to understand the impact of Sglt2 deletion on mineral and bone metabolism parameters in the settings of T2DM and CKD.

SGLT2 inhibitors are already changing the care for patients with CKD. Although further safety evaluation will be needed, results from the CREDENCE trial (https://clinicaltrials.gov/ct2/show/NCT02065791) are promising because there is no signal for increased risk of bone fracture even in patients with estimated glomerular filtration rate (eGFR) 30 to 90 mL/min/1.73 m^2^.^(^ ^33^ ^)^ Our findings from mice with an Sglt2 mutation fail to demonstrate any alterations in PTH, FGF23, and 1,25(OH)_2_D and that long‐term effects of the ubiquitous genetic deletion on bone health are relatively mild. Additional experimental studies in disease challenged animal models will be needed to further examine effects of Sglt2 inhibition in the setting of T2DM and CKD.

## Conflict of interest

SEQ has applied for patents related to therapeutic targeting of the ANGPT‐TEK pathway in ocular hypertension and glaucoma and receives research support, owns stock in and is a director of Mannin Research; in addition, SEQ is an external advisory board member of Astra Zeneca and receives consulting and advisory board fees from Roche and Janssen.

5

### Peer Review

The peer review history for this article is available at https://publons.com/publon/10.1002/jbm4.10526.

## Supporting information

Additional supporting information may be found online in the Supporting Information section.

**Figure S1** Sglt2 deletion does not impact potassium levels. Levels of serum potassium in WT and SP mice at 15 and 25 weeks of age. Values are expressed as mean ± SEM; *n* > 8 male mice/group. NS = not significant.

**Figure S2** Sglt2 deletion does not induce ketosis. Levels of serum ketones in WT and SP mice at 25 weeks of age. Values are expressed as mean ± SEM; *n* > 4 male mice/group. NS = not significant.

**Figure S3** Sglt2 deletion does not modify kidney 1,25(OH)_2_D metabolism, and bone GH/IGF1 and Wnt signaling. mRNA expression measured by quantitative RT‐PCR of (*A*) Cyp27b1 and Cyp24a1 in the entire kidney, (*B*) Igf1, Sost, Dkk1, and Axin2 in the tibia from 25‐week‐old WT and SP mice. Expression is normalized by Rpl19 mRNA expression in each sample. Values are expressed as mean ± SEM; *n* = 4–5 male mice/group. NS = not significant.
