# Supplementary figures and images for: Long‐Term Effects of Sglt2 Deletion on Bone and Mineral Metabolism in Mice
Source: JBMR Plus. 2021 Jul 6;5(8):e10526. doi: 10.1002/jbm4.10526 (PMC8328801; doi:10.1002/jbm4.10526)

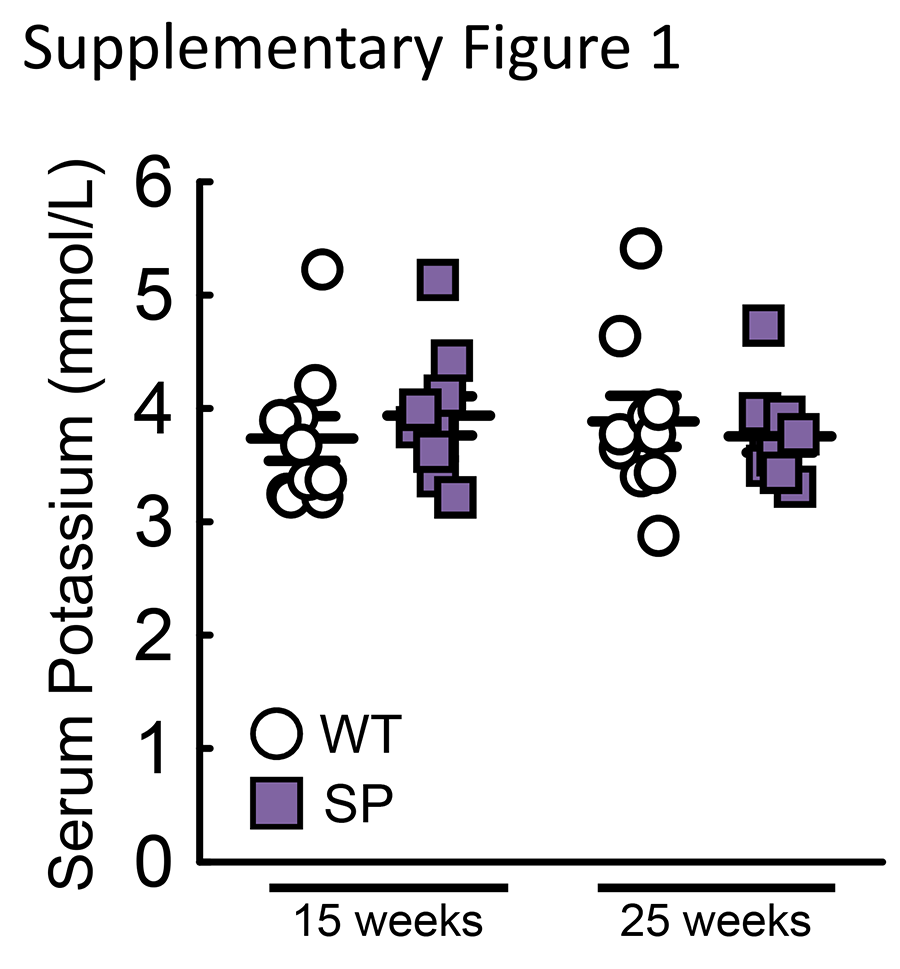

Supplement: Supplementary file 1 — Figure S1 Sglt2 deletion does not impact potassium levels. Levels of serum potassium in WT and SP mice at 15 and 25 weeks of age. Values are expressed as mean ± SEM; n > 8 male mice/group. NS = not significant. [file JBM4-5-e10526-s003.tif]

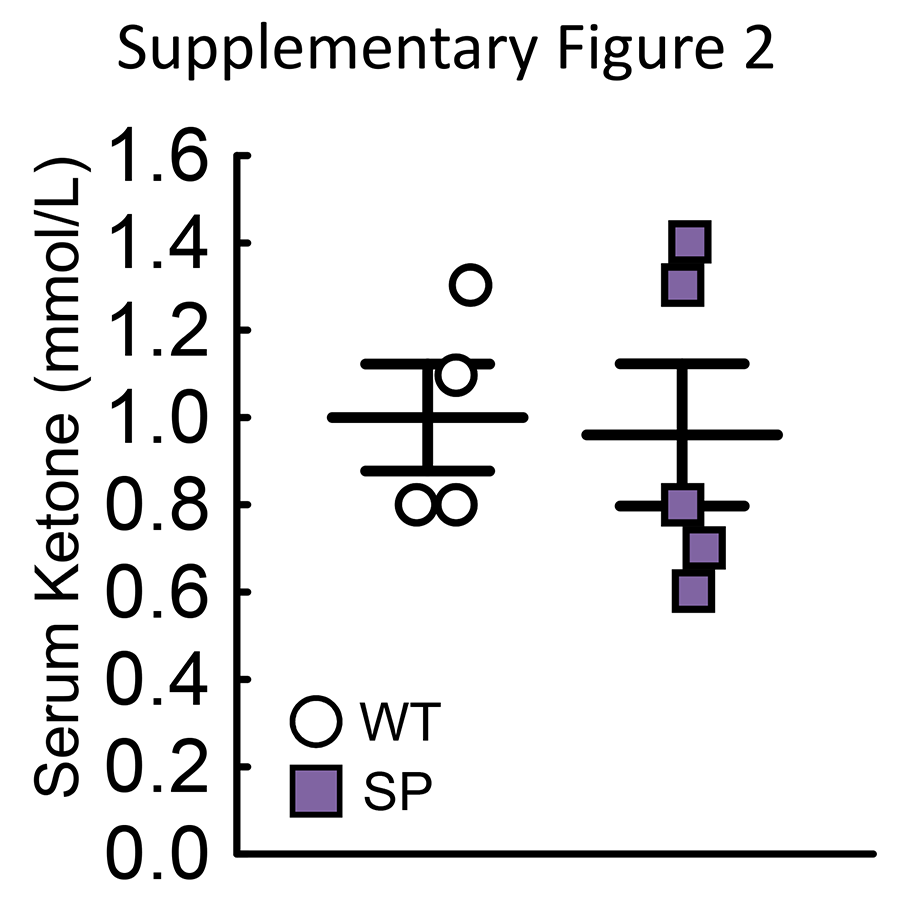

Supplement: Supplementary file 2 — Figure S2 Sglt2 deletion does not induce ketosis. Levels of serum ketones in WT and SP mice at 25 weeks of age. Values are expressed as mean ± SEM; n > 4 male mice/group. NS = not significant. [file JBM4-5-e10526-s001.tif]

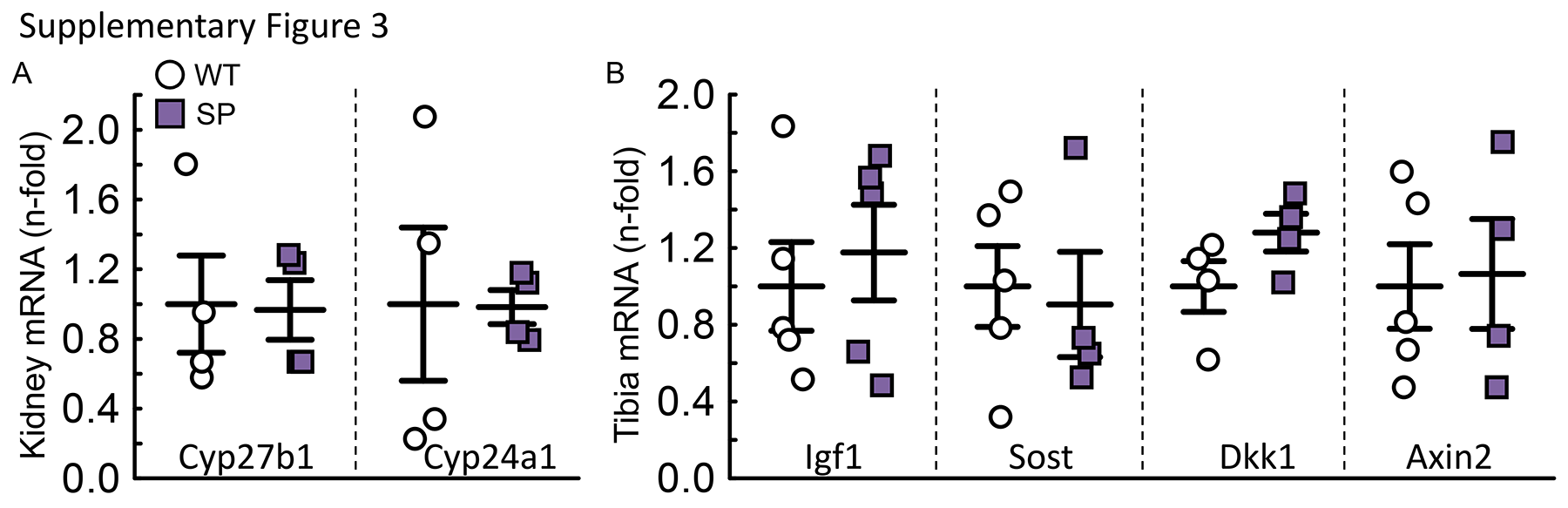

Supplement: Supplementary file 3 — Figure S3 Sglt2 deletion does not modify kidney 1,25(OH)2D metabolism, and bone GH/IGF1 and Wnt signaling. mRNA expression measured by quantitative RT‐PCR of (A) Cyp27b1 and Cyp24a1 in the entire kidney, (B) Igf1, Sost, Dkk1, and Axin2 in the tibia from 25‐week‐old WT and SP mice. Expression is normalized by Rpl19 mRNA expression in each sample. Values are expressed as mean ± SEM; n = 4–5 male mice/group. NS = not significant. [file JBM4-5-e10526-s002.tif]
